# Role of Obeticholic Acid, a Farnesoid X Receptor Agonist, in Nonalcoholic Fatty Liver Disease: A Systematic Review and Meta-analysis

**DOI:** 10.17925/EE.2024.20.2.8

**Published:** 2024-10-09

**Authors:** ABM Kamrul-Hasan, Sunetra Mondal, Lakshmi Nagendra, Thanikai Sasikanth, Afsar Ahammed, Shahin Ibn Rahman, Ashani Wickramarachchi, Naresh Parajuli, Saurav Khatiwada, Deep Dutta

**Affiliations:** 1. Department of Endocrinology, Mymensingh Medical College, Mymensingh, Bangladesh; 2. Department of Endocrinology, NRS Medical College, Kolkata, India; 3. Department of Endocrinology, JSS Medical College, JSS Academy of Higher Education and Research, Mysore, India; 4. Department of Endocrinology, National Hospital of Sri Lanka, Colombo, Sri Lanka; 5. Department of Endocrinology, National Institute of Traumatology & Orthopaedic Rehabilitation, Dhaka, Bangladesh; 6. Department of Endocrinology, BIRDEM General Hospital, Shahbag, Dhaka, Bangladesh; 7. Department of Endocrinology, University Medical Unit, National Hospital of Sri Lanka, Colombo, Sri Lanka; 8. Department of Medicine, Endocrine Unit, Tribhuvan University Institute of Medicine, Kathmandu, Nepal; 9. Department of Medicine, Endocrine Unit, Chitwan Medical College, Bharatpur, Chitwan, Nepal; 10. Department of Endocrinology, CEDAR Superspeciality Healthcare, Dwarka, New Delhi, India

**Keywords:** Farnesoid X nuclear receptor, hepatic transaminases, meta-analysis, metabolic dysfunction-associated fatty liver disease, nonalcoholic fatty liver disease, nonalcoholic steatohepatitis, obeticholic acid

## Abstract

**Background.** Obeticholic acid (OCA) has emerged as a promising drug in the management of nonalcoholic fatty liver disease (NAFLD). This meta-analysis aimed to analyse the therapeutic effect of OCA on NAFLD. **Methods.** Randomized controlled trials (RCTs) involving patients with NAFLD receiving OCA in the intervention arm and placebo in the control arm were searched throughout the electronic databases. The primary outcomes were changes in non-invasive markers of hepatic fibrosis and liver histology. The secondary outcomes included changes in liver enzymes, metabolic parameters from baseline and adverse events (AEs). **Results.** Four RCTs involving 1,278 subjects met the inclusion criteria. Over 6 weeks to 18 months of clinical use, OCA outperformed placebo in resolving definite nonalcoholic steatohepatitis (odds ratio [OR] 1.60, 95% confidence interval [CI] [1.04–2.48], p=0.03) and improving fibrosis (OR 2.23, 95% CI [1.56–3.20], p<0.0001), hepatocellular ballooning (OR 1.83, 95% CI [1.35–2.47], p<0.0001) and lobular inflammation (OR 1.62, 95% CI [1.13–2.32], p=0.009). OCA did not improve the enhanced liver fibrosis score and steatosis better than placebo, and demonstrated superior efficacy compared with the placebo in reducing serum alanine aminotransferase, aspartate aminotransferase and gamma-glutamyl transferase levels. Although a favourable effect of OCA over placebo was seen in body-weight reduction, the OCA use was associated with adverse changes in lipid parameters. Except for the greater risk of pruritus and constipation, the AE profile was comparable between the OCA and placebo groups. **Conclusions.** OCA has a favourable efficacy in improving liver histology and liver enzymes. However, the worsening of lipid parameters and other AEs with the OCA use warrants further investigation.

Hepatic steatosis is the liver manifestation of metabolic syndrome and a common cause of chronic liver disease. Nonalcoholic fatty liver disease (NAFLD) diagnosis relies on the presence of hepatic steatosis, defined as >5% fat accumulation in the liver, as observed through imaging or histology. This diagnosis is made when there are no other concurrent secondary causes, such as significant alcohol use.^1^ Nonalcoholic steatohepatitis (NASH) is a distinct condition characterized by the occurrence of inflammation, damage to hepatocytes and fibrosis in individuals with hepatic steatosis.^1^ A recent proposal suggested renaming the condition as metabolic dysfunction-associated steatotic liver disease (MASLD).^2^ The criterion for MASLD uses the same standard for hepatic steatosis but identifies metabolic dysregulatory factors as a prerequisite for the diagnosis to be entertained. MASLD is diagnosed in a patient with evidence of hepatic steatosis and having at least one of the following conditions: overweight/obesity, type 2 diabetes (T2D) or signs of metabolic dysfunction.^2^ The new term MASLD provides a more precise understanding of the underlying mechanisms of fatty liver disease in overweight or obese people who have T2D or metabolic syndrome.^2^ The most significant difference between NAFLD and the diagnosis of MASLD, however, is not the formal recognition of metabolic dysregulatory pathways in the development of the disease, but rather the removal of exclusion of concurrent liver disease to entertain the diagnosis.^2^

With the growing global pandemic of obesity and the associated metabolic syndrome, the global prevalence of NAFLD has risen from 2 5.5% in or before 2005 to 38% in or after 2016.^3^ Asian people exhibit higher insulin resistance than Westerners for a given body mass index.^4^ They also have considerable heterogeneity in the prevalence of NAFLD across countries and are on a rising trend. More men are affected than women. T2D is a strong independent risk factor for the progression of NAFLD into steatohepatitis and cirrhosis.^4^

Management of NAFLD consists of lifestyle interventions to achieve weight reduction, intensive cardiovascular risk factor modification and liver-directed pharmacotherapy. Several anti-diabetic, anti-obesity and lipid-modifying medications, as well as vitamin supplementations and innovational drugs, have been tested in trials for the management of NAFLD.^5^ Obeticholic acid (OCA) (also known as 6α-ethyl-chenodeoxycholic acid) is a semi-synthetic bile acid derivative and a potent activator of farnesoid X nuclear receptor (FXR) in the liver and intestine, leading to increased release of fibroblast growth factor (FGF)-19 from the ileum.^5^ This forms a complex with the FGF receptor, which inhibits bile acid synthesis via inhibition of cholesterol-7α -hydroxylase (CYP7A1) and facilitates bile acid excretion from hepatocytes, thus reducing the hepatic burden of toxins.^5,6^ Furthermore, it suppresses transforming growth factor-β, hepatic stellate cells, extracellular matrix proliferation and inflammatory cell infiltration; thus, it reduces liver steatosis, inflammation and fibrosis in animal models.^7^ Moreover, when bound to the FXR, lipophilic bile acids improve hepatic insulin sensitivity and decrease hepatic lipid synthesis, providing beneficial effects against metabolic syndrome.^6^ Its use is currently approved in patients with primary biliary cirrhosis (PBC) not tolerating ursodeoxycholic acid.^8,9^

Although the use of OCA in NAFLD has not yet been approved, it has a promising role in treating these patients.^10^ Few clinical trials investigating the use of OCA in NAFLD are available, with variable efficacy and safety outcomes. Some meta-analyses were also conducted, but all of them reported the effects of OCA with combined analysis on diverse clinical conditions, including PBC and NAFLD.^5,11^ Moreover, those meta-analyses did not include recent clinical trials. With this background, this meta-analysis aimed to evaluate the efficacy and safety of OCA in patients with NAFLD.

## Methods

The meta-analysis rigorously followed the Cochrane Handbook for Systematic Reviews of Interventions protocols.^12^ This study was registered with PROSPERO and assigned the registration number CRD12024497735. The study adopted the Preferred Reporting Items for Systematic reviews and Meta-Analyses guidelines to guarantee transparent and rigorous reporting of methods and results; the specific information can be found in the supplementary material.^12^ No additional ethical approval was required for this review, as all randomized controlled trials (RCTs) already had their respective approvals.

A thorough investigation was conducted by searching multiple databases and registers, such as MEDLINE (via PubMed), Scopus, Google Scholar, Cochrane Central Register, International Clinical Trials Registry Platform and ClinicalTrials.gov. The search covered these sources’ inception until 25 November 2023. The search strategy used a Boolean approach with the terms ‘((obeticholic acid) OR (INT-747) OR (six alpha-ethyl-chenodeoxycholic acid)) AND ((fatty liver) OR (nonalcoholic fatty liver disease) OR (nonalcoholic steatohepatitis) OR (metabolic dysfunction-associated steatotic liver disease) OR (metabolic dysfunction-associated fatty liver disease) OR (metabolic dysfunction–associated steatohepatitis))’; the search terms were applied to titles only. A thorough and careful search was conducted to find any recently published or unpublished clinical trials in English. This search included examining references within previous meta-analyses, the RCTs included in this study and relevant journals.

The selection of studies for this meta-analysis was based on the Population, Intervention, Comparison, Outcomes and Study criteria. The eligible studies necessitated a minimum of two treatment arms or groups, wherein one group received daily doses of OCA at either 10 or 25 mg, and the other group received a placebo, all in patients diagnosed with NAFLD. The primary outcomes were the changes in non-i nvasive markers of hepatic fibrosis and liver histology from baseline. The secondary outcomes encompassed changes in liver enzymes, synthetic liver functions, metabolic parameters, lipid profile from baseline and adverse events (AEs).

Data extraction was independently conducted by six review authors using standardized data extraction forms, with details provided elsewhere.^13^ The handling of missing data has also been elaborated upon in the same source.^13^ Six authors independently performed the risk of bias (RoB) assessment using the RoB tool in the Review Manager (RevMan) computer program, version 7.2.0.^14^ Specific biases have been outlined in the same source.^13^

For analysis, the International System of Units (SI units) were used for the variables. The data were aggregated using random-e ffect models to analyse the primary and secondary outcomes. RevMan was used to compare the primary and secondary outcomes between the OCA (experimental drug) and control groups in the included studies. The results were expressed as mean differences (MDs) for continuous outcomes and odds ratios (ORs) or risk ratios for categorical outcomes with 95% confidence intervals (CIs). Forest plots created using RevMan portrayed outcomes, with the left side favouring OCA and the right side favouring placebo. A significance level of p<0.05 was used. The results included forest plots incorporating the data from at least two RCTs.

The evaluation of heterogeneity was initially performed by analysing forest plots. Afterwards, a chi-square test was conducted with N-1 degrees of freedom and a significance level of 0.05 to ascertain the statistical significance. Additionally, the *I*^2^ test was used in further analysis.^15^ The details of interpreting *I*^2^ values have already been elaborated elsewhere.^13^

The Grading of Recommendations Assessment, Development and Evaluation methodology was used to assess the quality of evidence pertaining to each outcome of the meta-analysis.^16^ The process of creating the summary of findings (SoF) table and assessing the quality of evidence as ‘high’, ‘moderate’, ‘low’ or ‘extremely low’ has previously been described.^13^ Publication bias was evaluated using funnel plots, in which studies falling outside the inverted funnel plot indicated the presence of substantial publication bias.^17^

## Results

### Search results

**Figure 1:**
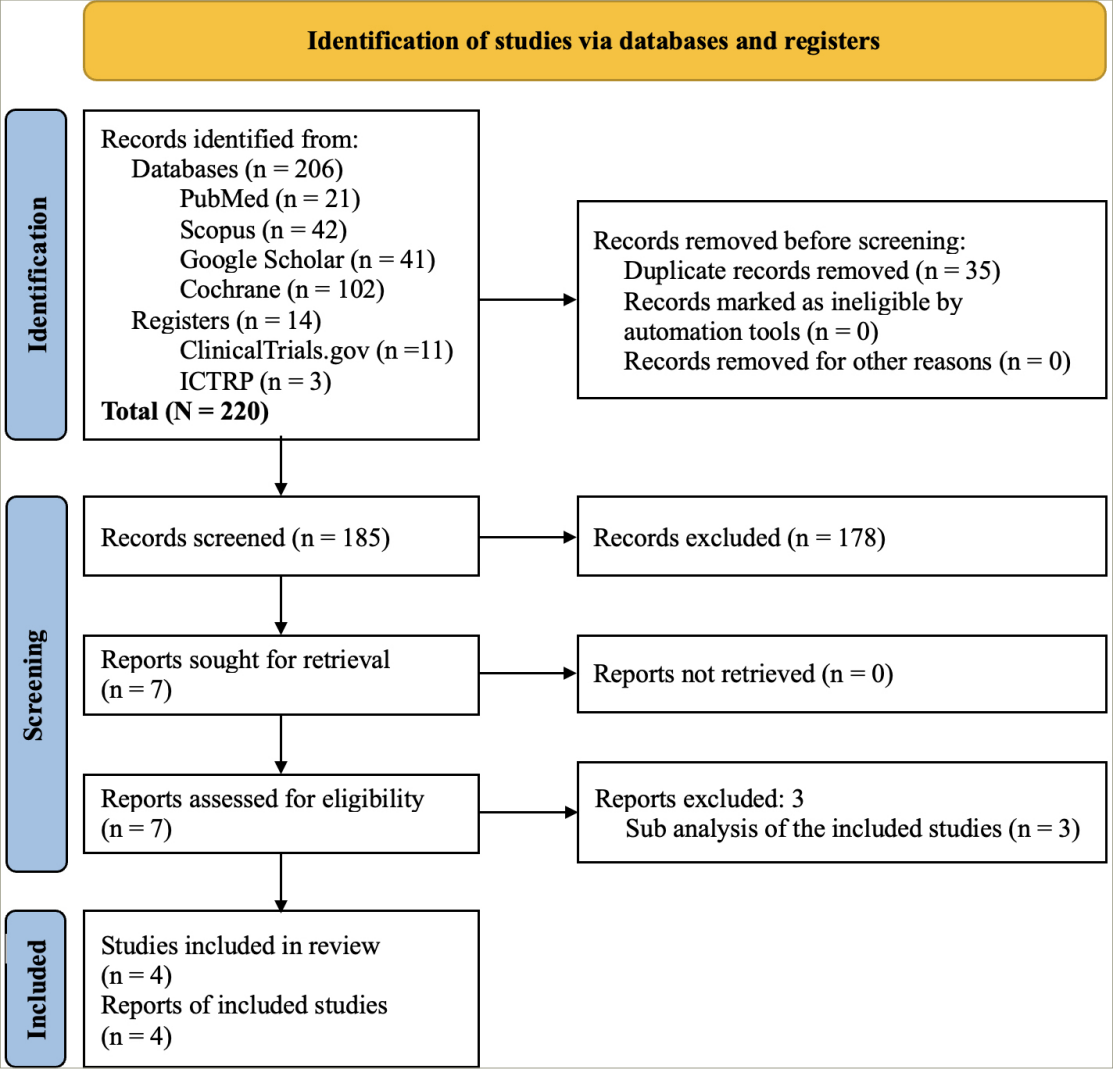
Flow chart of study retrieval and inclusion in the meta-analysis

The study selection process is depicted in *Figure 1*. A total of 220 articles were found after the initial search. After screening the titles and abstracts, followed by full texts, the search was reduced to seven studies, evaluated in detail for inclusion in this meta-analysis. Four studies that fulfilled all criteria involving 1,278 subjects were analysed in this meta-analysis.^18–21^ Three studies were excluded because they were subanalyses of the included studies.^22–24^

*ICTRP = International Clinical Trials Registry Platform.*

### Study characteristics

In all four included RCTs, the study subjects received either experimental (OCA) or control (placebo) drugs. The dose of OCA ranged from 10 to 50 g/day. One study spanned 6 weeks, one 72 weeks and two 18 months.^20,21^ The details of the included and excluded studies are shown in *Supplementary Table S1* and *Supplementary Table S2*, respectively.

### Risk of bias in the included randomized controlled trials

The RoB across the 10 studies included in the meta-analysis is illustrated in *Supplementary Figure S1*. Every study (100%) demonstrated low risks concerning all types of bias except ‘other bias’, which was present in all (100%). The comprehensive RoB assessment process is provided as a supplementary file (*Supplementary Table S3*). Publication bias was assessed through funnel plots given in *Supplementary Figure S2*.

### Effect of obeticholic acid on markers of hepatic fibrosis

#### Enhanced liver fibrosis score

**Figure 2:**
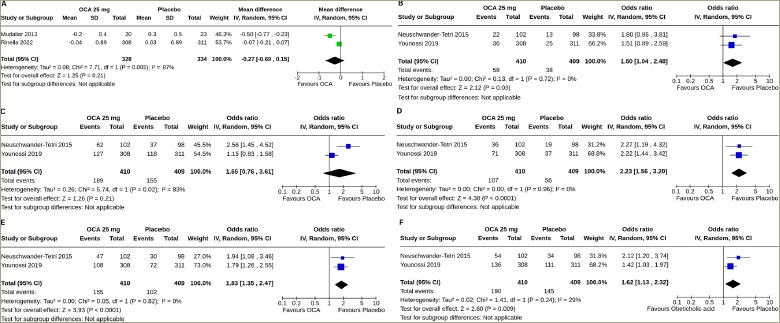
Impact on enhanced liver fibrosis score, definite non-alcoholic steatohepatitis, steatosis, fibrosis, hepatocellular ballooning and lobular inflammation

Data from two studies involving 662 subjects (OCA 328 and placebo 334) with NAFLD were analysed to determine the impact of OCA on enhanced liver fibrosis (ELF) scores.^18,20^ Changes in the ELF score from the baseline were similar in the OCA group and the control group (MD -0.27, 95% CI [-0.69 to 0.15], p=0.21, *I*^2^=21% [not an important heterogeneity], high certainty of evidence) (*Figure 2A*).

#### Effect of obeticholic acid on liver histology

Data from two studies involving 819 subjects (OCA 410 and placebo 409) with NAFLD were analysed to determine the impact of OCA on the resolution of definite NASH and improvements in steatosis, fibrosis, hepatocellular ballooning and lobular inflammation.^19,21^ A higher proportion of the subjects with OCA than placebo had a resolution of definite NASH (OR 1.60, 95% CI [1.04–2.48], p=0.03, *I*^2^=0% [not an important heterogeneity], high certainty of evidence) (*Figure 2B*), improvements in fibrosis (OR 2.23, 95% CI [1.56–3.20], p<0.0001, *I*^2^=0% [not an important heterogeneity], high certainty of evidence) (*Figure 2D*), improvements in hepatocellular ballooning (OR 1.83, 95% CI [1.35–2.47], p<0.0001, *I*^2^=0% [not an important heterogeneity], high certainty of evidence) (*Figure 2E*) and improvements in lobular inflammation (OR 1.62, 95% CI [1.13–2.32], p=0.009, *I*^2^=29% [mild heterogeneity], high certainty of evidence) (*Figure 2F*). OCA was similarly effective in the improvement in steatosis (OR 1.65, 95% CI [0.76–3.61], p=0.21, *I*^2^=83% [high heterogeneity], high certainty of evidence) (*Figure 2C*).

*Forest plot highlighting the impact of obeticholic acid and placebo on (A) changes in the enhanced liver fibrosis score, (B) resolution of definite nonalcoholic steatohepatitis, (C) improvement in steatosis, (D) improvement in fibrosis, (E) improvement in hepatocellular ballooning and (F) improvement in lobular inflammation from baseline.*

*CI = confidence interval; df = degrees of freedom; IV = intravenous; OCA = obeticholic acid; SD = standard deviation.*

### Effect of obeticholic acid on hepatic enzymes and functions

#### Alanine aminotransferase

**Figure 3:**
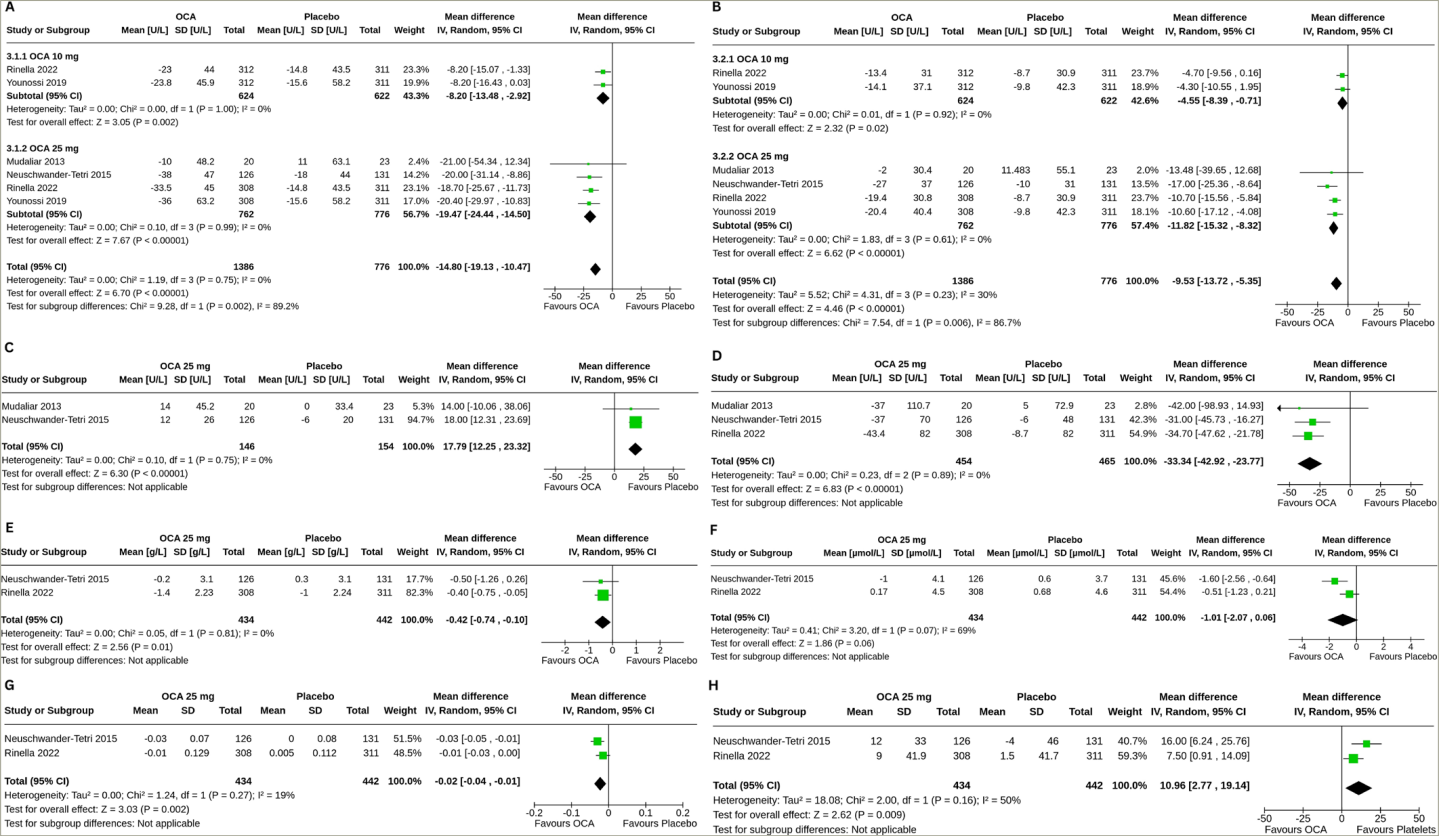
Impact on alanine aminotransferase, aspartate aminotransferase, alkaline phosphatase, gamma-glutamyl transferase, albumin, bilirubin, international normalized ratio and platelet

Two studies (1,246 subjects: OCA 624 and placebo 622) with OCA 10 mg and four studies (1,538 subjects: OCA 762 and placebo 776) with OCA 25 mg reported the results of alanine aminotransferase (ALT) changes from baseline to the end of the trial.^18–21^ Compared with the placebo, both OCA 10 mg (MD -8.20 U/L, 95% CI [-13.48 to -2.92], p=0.002, *I*^2^=0% [not an important heterogeneity]) and OCA 25 mg (MD -19.47 U/L, 95% CI [-24.44 to -14.50], p<0.00001, *I*^2^=0% [not an important heterogeneity], moderate certainty of evidence) achieved greater reductions in ALT. A 25 mg dose of OCA reduced ALT to more than 10 mg of OCA (p=0.002) (*Figure 3A*).

#### Aspartate aminotransferase

Two studies (1,246 subjects: OCA 624 and placebo 622) with OCA 10 mg and four studies (1,538 subjects: OCA 762 and placebo 776) with OCA 25 mg reported the results of aspartate aminotransferase (AST) changes from baseline to the end of the trial.^18–21^ Compared with the placebo, both OCA 10 mg (MD -4.55 U/L, 95% CI [-8.39 to -0.71], p=0.02, *I*^2^=0% [not an important heterogeneity]) and OCA 25 mg (MD -11.82 U/L, 95% CI [-15.32 to -8.32], p<0.00001, *I*^2^=0% [not an important heterogeneity], moderate certainty of evidence) achieved greater reductions in AST. A 25 mg dose of OCA reduced AST to more than 10 mg of OCA (p=0.0006) (*Figure 3B*).

#### Alkaline phosphatase

Two studies (300 subjects: OCA 146 and placebo 154) with OCA 25 mg reported the results of alkaline phosphatase (ALP) changes from baseline to the end of the trial.^18,19^ Compared with the placebo, a greater increase in ALP was observed with OCA 25 mg (MD 17.79 U/L, 95% CI [12.25 to 23.32], p<0.00001, *I*^2^=0% [not an important heterogeneity], high certainty of evidence) (*Figure 3C*).

#### Gamma-glutamyl transferase

Three studies (919 subjects: OCA 454 and placebo 465) with OCA 25 mg reported the results of gamma-glutamyl transferase (GTT) changes from baseline to the end of the trial.^18–20^ Compared with the placebo, a greater reduction in gamma-glutamyl transferase (GGT) was achieved with OCA 25 mg (MD -33.34 U/L, 95% CI [-42.92 to -23.77], p<0.00001, *I*^2^=0% [not an important heterogeneity], moderate certainty of evidence) (*Figure 3D*).

#### Albumin

Two studies (876 subjects: OCA 434 and placebo 442) with OCA 25 mg reported the results of serum albumin changes from baseline to the end of the trial.^19,20^ Compared with the placebo, a greater reduction in serum albumin was observed with OCA 25 mg (MD -0.42 g/L, 95% CI [-0.74 to -0.10], p=0.01, *I*^2^=0% [not an important heterogeneity], high certainty of evidence) (*Figure 3E*).

#### Bilirubin

Two studies (876 subjects: OCA 434 and placebo 442) with OCA 25 mg reported the results of serum bilirubin changes from baseline to the end of the trial.^19,20^ Changes in serum bilirubin were similar in the OCA 25 mg and the placebo groups (MD -1.01 μmol/L, 95% CI [-2.07 to 0.06], p=0.06, *I*^2^=69% [moderate heterogeneity], moderate certainty of evidence) (*Figure 3F*).

*Forest plot highlighting the effects of obeticholic acid and placebo on changes in (A) alanine aminotransferase, (B) aspartate aminotransferase, (C) alkaline phosphatase, (D) gamma-glutamyl transferase, (E) albumin, (F) bilirubin, (G) international normalized ratio and (H) platelet from baseline.*

*CI = confidence interval; df = degrees of freedom; IV = intravenous; OCA = obeticholic acid; SD = standard deviation.*

#### International normalized ratio

Two studies (876 subjects: OCA 434 and placebo 442) with OCA 25 mg reported the results of international normalized ratio (INR) changes from baseline to the end of the trial.^19,20^ Compared with the placebo, a greater reduction in INR was observed with OCA 25 mg (MD -0.02, 95% CI [-0.04 to -0.01], p=0.002, *I*^2^=19% [not an important heterogeneity], high certainty of evidence) (*Figure 3G*).

### Effect of obeticholic acid on platelet count

Two studies (876 subjects: OCA 434 and placebo 442) with OCA 25 mg reported the results of changes in platelet count from baseline to the end of the trial.^19,20^ Compared with the placebo, a greater increase in platelet count was observed with OCA 25 mg (MD 10.96 x 10^9^ /L, 95% CI [2.77–19.14], p=0.009, *I*^2^=50% [moderate heterogeneity], moderate certainty of evidence) (*Figure 3H*).

### Effect of obeticholic acid on lipid profile

**Figure 4:**
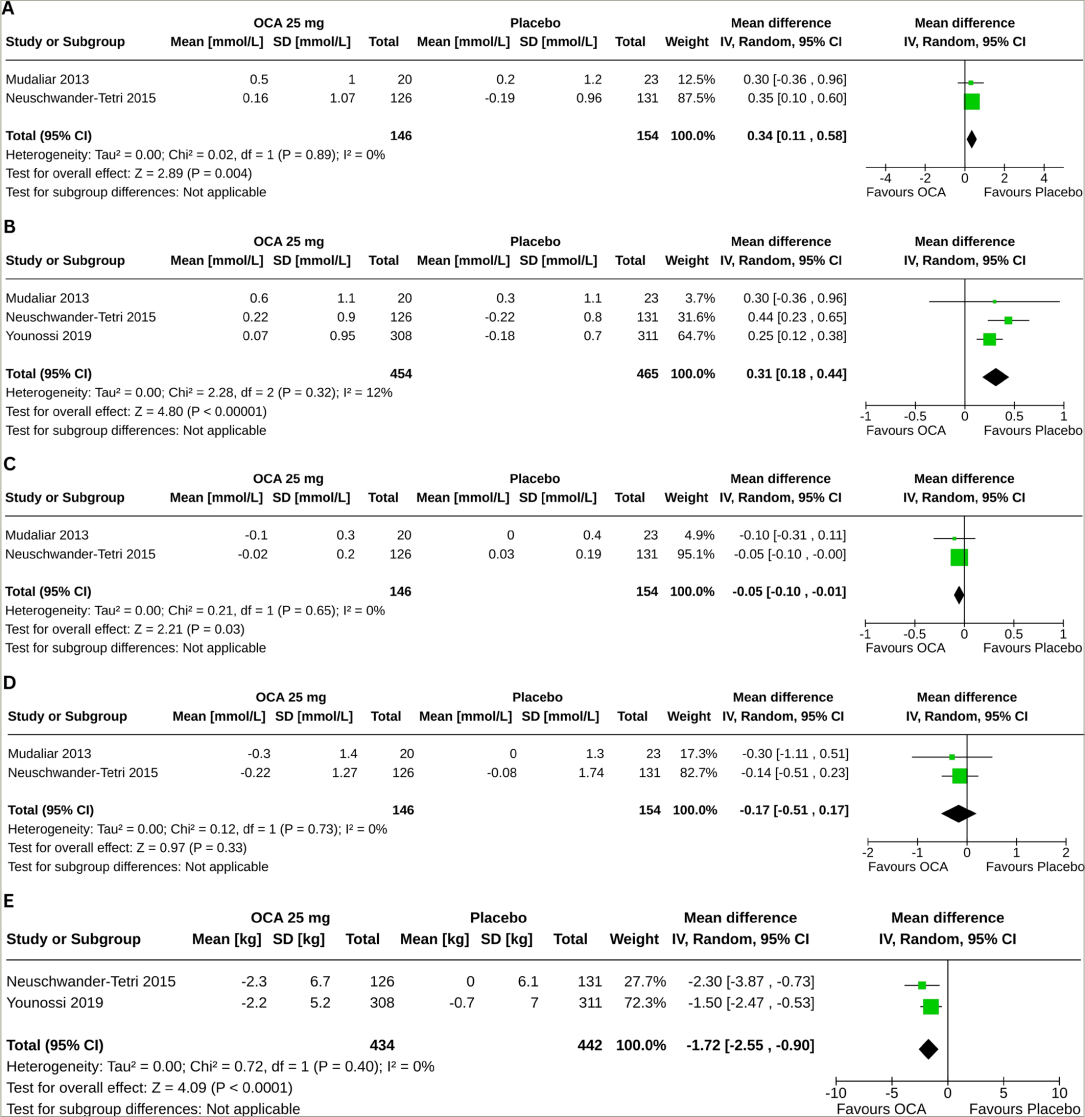
Impact on total, low-and high-density lipoprotein cholesterol, triglyceride and body weight

Two studies (300 subjects: OCA 146 and placebo 154) with OCA 25 mg reported the results of changes in total cholesterol (TC), high-density lipoprotein cholesterol (HDL-C) and triglyceride (TG), and three studies (919 subjects: OCA 454 and placebo 465) with OCA 25 mg reported the results of changes in low-density lipoprotein cholesterol (LDL-C) from baseline to the end of the trial.^18,19,21^ Compared with the placebo, greater increases in TC (MD 0.34 mmol/L, 95% CI [0.11–0.58], p=0.004, *I*^2^=0% [not an important heterogeneity], high certainty of evidence) (*Figure 4A*) and LDL-C (MD 0.31 mmol/L, 95% CI 0.18–0.44], p<0.00001, *I*^2^=12% [not an important heterogeneity], moderate certainty of evidence) (*Figure 4B*) were observed with OCA 25 mg. The OCA 25 mg group also had a greater reduction in HDL-C (MD -0.05 mmol/L, 95% CI [-0.10 to -0.01], p=0.03, *I*^2^=0% [not an important heterogeneity], moderate certainty of evidence) (*Figure 4C*); changes in TG were similar in the two groups (MD -0.17 mmol/L, 95% CI [-0.51 to 0.17], p=0.33, *I*^2^=0% [not an important heterogeneity], high certainty of evidence) (*Figure 4D*).

### Effect of obeticholic acid on body weight

Two studies (876 subjects: OCA 434 and placebo 442) with OCA 25 mg reported the results of body weight changes from baseline to the end of the trial.^19,21^ Compared with the placebo, a greater reduction in body weight was observed with OCA 25 mg (MD -1.72 kg, 95% CI [-2.55 to -0.90], p<0.0001, *I*^2^=0% [not an important heterogeneity], high certainty of evidence; *Figure 4E*).

### Safety parameters

*Table 1* describes the results of safety outcomes in the meta-analysis. Compared with the placebo, OCA was associated with higher risks of pruritus (any) (OR 4.02, 95% CI [2.16–7.50], p<0.0001, *I*^2^=47% [mild heterogeneity], moderate certainty of evidence), pruritus (grade 1) (OR 1.81, 95% CI [1.37–2.39], p<0.0001, *I*^2^=0% [not an important heterogeneity], moderate certainty of evidence), pruritus (grade 2) (OR 6.44, 95% CI [4.37–9.49], p<0.0001, *I*^2^=0% [not an important heterogeneity], moderate certainty of evidence), pruritus (grade 3) (OR 11.69, 95% CI [3.89–35.07], p<0.0001, *I*^2^=0% [not an important heterogeneity], high certainty of evidence) and constipation (OR 2.12, 95% CI [1.41–3.20], p=0.0003, *I*^2^=0% [not an important heterogeneity], moderate certainty of evidence). Headache risk was lower with OCA (OR 0.63, 95% CI [0.41–0.97], p=0.04, *I*^2^=0% [not an important heterogeneity]). Treatment-related AEs, withdrawal due to an AE, serious AEs, deaths, nausea, vomiting and diarrhoea, abdominal pain, urinary and upper respiratory tract infections, nasopharyngitis and dizziness or syncope were similar in the two groups.

*Forest plot highlighting the impact of obeticholic acid and placebo on changes in (A) total cholesterol, (B) low-density lipoprotein cholesterol, (C) high-density lipoprotein cholesterol, (D) triglyceride and (E) body weight from baseline.*

*CI = confidence interval; df = degrees of freedom; IV = intravenous; OCA = obeticholic acid; SD = standard deviation.*

### Grading of the results

The grades of the certainty of evidence of the results are given in the SoF table (*Supplementary Table S4*).

## Discussion

The present meta-analysis incorporated the results of RCTs of OCA in NAFLD published to date. It highlights the efficacy and safety of OCA in managing NAFLD compared with a placebo. OCA was more effective than placebo in improving liver histology and reducing liver enzymes and body weight. Potential concerns with OCA use were the greater increments in TC and LDL-C and higher risks of pruritus and constipation.

Over 6 weeks to 18 months of clinical use, OCA was not superior to placebo in improving the ELF score, a non-i nvasive marker of liver fibrosis, although a histological improvement in fibrosis was observed. However, this observation is limited by the fact that one of the two ELF score-reporting studies was only of 6-week duration, which may not be long enough to see an effect of OCA on the ELF score.^18^ Sustained reductions in FibroTest scores, another proprietary serum fibrosis marker, were observed in OCA-treated patients compared with a small increase among placebo-treated patients in the study by Rinella et al.^20^ With OCA, Rinella et al. reported reductions in the Fibrosis-4 and AST to Platelet Ratio Index scores, the composite fibrosis scores based on clinical parameters. Moreover, a dose-dependent decrease in liver stiffness (assessed by FibroScan®, Echosens, Paris, France) was observed in patients treated with OCA; all these parameters worsened with the placebo.^20^ This meta-analysis also found the superiority of OCA over placebo in the resolution of definite NASH and improvements in hepatocellular ballooning and lobular inflammation. Neuschwander-Tetri et al. also found greater reductions in the fibrosis score, the total NAFLD score, the hepatocellular ballooning score and the lobular inflammation score with OCA than with the placebo.^19^ In this meta-analysis, OCA and placebo performed similarly in steatosis improvement, although Neuschwander-Tetri et al. observed greater reductions in the steatosis score in their study.^19^

**Table 1:** The results of safety outcomes in meta-analyses

		Number of participants with outcomes/participants analysed (n)			
Safety variables	Number of RCTs		Obeticholic acid arm	Placebo arm	*I*^2^ (%)	Pooled effect size, OR [95% CI]	p
Treatment-related AEs	2	602/678	554/680	81	0.71 [0.06–9.06]	0.79
Withdrawal due to an AE	2	83/678	42/680	12	1.91 [0.78–4.67]	0.15
Serious AEs	2	93/678	75/680	NA	1.28 [0.92–1.77]	0.14
Deaths	2	1/678	2/680	NA	0.50 [0.05–5.51]	0.57
Pruritus (any)	3	369/819	134/822	47	4.02 [2.16–7.50]	<0.0001
Pruritus (grade 1)	2	157/799	96/799	0	1.81 [1.37–2.39]	<0.0001
Pruritus (grade 2)	2	173/799	33/799	0	6.44 [4.37–9.49]	<0.0001
Pruritus (grade 3)	2	39/799	3/799	0	11.69 [3.89–35.07]	<0.0001
Nausea, vomiting and diarrhoea	3	188/819	203/822	0	0.90 [0.72–1.14]	0.39
Abdominal pain	2	119/799	106/799	0	1.15 [0.86–1.52]	0.35
Constipation	3	75/819	37/822	0	2.12 [1.41–3.20]	0.0003
Urinary tract infection	2	64/799	52/799	0	1.25 [0.85–1.84]	0.25
Upper respiratory tract infection	2	54/678	46/680	20	1.02 [0.34–3.08]	0.98
Nasopharyngitis	2	45/678	43/680	8	1.00 [0.48–2.12]	0.99
Dizziness or syncope	2	28/799	32/799	0	0.87 [0.52–1.46]	0.60
Headache	3	37/819	57/822	0	0.63 [0.41–0.97]	0.04

*AEs = adverse events; CI = confidence interval; NA = not applicable; OR = odds ratio; RCTs = randomized controlled trials.*

Besides demonstrating consistent enhancements in histological aspects, OCA outperformed the placebo in enhancing liver well-being, as evidenced by meaningful, dose-dependent decreases in indicators of liver injury (ALT and AST) and oxidative stress (GGT). The slight increases in ALP align with prior observations in individuals with NAFLD and could be linked to the on-target impact of FXR activation.^25^

The OCA use may hamper synthetic functions of the liver, as evidenced by greater reductions in serum albumin and INR in this meta-analysis. Serum albumin and bilirubin remained unchanged in patients with PBC throughout a clinical trial.^26^ More evidence from large studies is needed to confirm such effects of OCA on hepatic synthetic functions.

This meta-analysis found decremental effects of OCA on lipid profile in patients with NAFLD, with greater increases in TC and LDL-C and reductions in HDL-C than placebo. The changes in TG were similar in the two groups. The cardiovascular AEs were infrequent and comparable across the OCA and placebo groups.^19,21^ Long-term studies are needed to determine the clinical significance of the changes in lipid parameters induced by OCA. In a recent meta-analysis, OCA was associated with a greater increase in LDL-C and a reduction in TG and HDL-C than placebo in patients with PBC, whereas similar changes were observed in TG.^11^ Weight reduction, another metabolic parameter, was more pronounced with OCA than with the placebo. The positive influence of OCA on weight is crucial, especially considering the widespread presence of obesity and metabolic irregularities in this group, coupled with the confirmed effectiveness of weight loss in altering the progression of NAFLD. OCA was generally well tolerated, according to the result of this meta-analysis. Pruritus, especially in more severe forms, was remarkably higher with OCA than with the placebo. A higher risk of pruritus is also observed in OCA-treated patients with PBC.^11,27^ The pathogenesis of pruritus remains unclear. As pruritus induction has been observed in all clinical trials testing other FXR agonists, pruritus appears to be an FXR-mediated class effect rather than a specific adverse effect unique to OCA.^11,28^ Other than a higher risk for constipation, gastrointestinal AEs were also not increased with OCA.

### Strengths and limitations of the meta-analysis

The key strength lies in being the first systematic review and meta-analysis specifically dedicated to evaluating the effects of OCA in individuals with NAFLD. Nevertheless, several limitations are noteworthy. The scarcity of eligible RCTs may have compromised the robustness of our conclusions. Certain included RCTs exhibited relatively small sample sizes, potentially impacting the statistical power and precision of our findings. We could not analyse the effect of OCA 10 mg due to the lack of availability of an adequate number of studies for meta-analysis. Another constraint arises from inadequate outcome measures in the available literature, necessitating the utilization of estimated values for quantitative analysis, thereby introducing a potential source of distortion in the results. Variations in baseline characteristics among participants in the included studies, including comorbidities and medications, may have influenced overall outcomes.

## Conclusion

OCA showed favourable liver histology and biochemistry outcomes in patients with NAFLD. However, it has also shown some AEs, including pruritus and worsening lipid profile, whose long-term impact warrants evaluation in a larger study with a longer duration of follow-up.
